# Exploring Addictive Online Behaviors in Patients with Narcolepsy Type 1

**DOI:** 10.3390/healthcare10112169

**Published:** 2022-10-30

**Authors:** Giorgia Varallo, Alessandro Musetti, Anita D’Anselmo, Alessio Gori, Emanuele Maria Giusti, Fabio Pizza, Gianluca Castelnuovo, Giuseppe Plazzi, Christian Franceschini

**Affiliations:** 1 Department of Medicine and Surgery, University of Parma, 43125 Parma, Italy; 2 Department of Humanities, Social Sciences and Cultural Industries, University of Parma, 43121 Parma, Italy; 3 Department of Biomedical and Neuromotor Sciences (DIBINEM), University of Bologna, 40123 Bologna, Italy; 4 Department of Health Sciences, University of Florence, Via di San Salvi 12, Pad. 26, 50135 Florence, Italy; 5 Integrated Psychodynamic Psychotherapy Institute (IPPI), 50122 Florence, Italy; 6 Psychology Research Laboratory, IRCCS Istituto Auxologico Italiano, Ospedale San Luca, 20149 Milan, Italy; 7 Department of Psychology, Catholic University of Milan, 20123 Milan, Italy; 8 IRCCS Istituto delle Scienze Neurologiche di Bologna (ISNB), 40139 Bologna, Italy; 9 Psychology Research Laboratory, IRCCS Istituto Auxologico Italiano, Ospedale San Giuseppe, 28824 Verbania, Italy; 10 Department of Biomedical, Metabolic and Neural Sciences, University of Modena and Reggio Emilia, 41125 Modena, Italy

**Keywords:** narcolepsy type 1, sleep, addictive behaviors, compulsive Internet use, problematic social media use, problematic online gaming, clinical psychology

## Abstract

Background: Narcolepsy type 1 (NT1) is a rare neurological sleep disorder caused by the loss of neurons that produce hypocretin—a peptide that plays a crucial role in addictive behaviors. We aimed to compare, for the first time, levels of problematic online gaming, problematic social media use, and compulsive Internet use between NT1 patients and healthy controls (HC), and to evaluate the association between anxiety, depression, and emotion dysregulation with addictive online behaviors in NT1 patients. Methods: A total of 43 patients with NT1 and 86 sex- and age-matched HC participated in an online cross-sectional survey. Results: NT1 patients did not differ from HC in terms of problematic social media use and compulsive Internet use but displayed higher levels of problematic online gaming compared to HC. Higher levels of emotion dysregulation were significantly associated with higher levels of problematic social media use and compulsive Internet use, while none of the tested factors were associated with problematic online gaming. Conclusion: NT1 patients and HC had similar levels of problematic social media use and compulsive Internet use, but NT1 patients showed higher levels of problematic online gaming. Emotion dysregulation might be an intervention target for reducing compulsive Internet use and problematic social media use.

## 1. Introduction

Narcolepsy type 1 (NT1) is a chronic neurological disorder marked by clinical manifestations such as excessive daytime sleepiness (EDS), cataplexy (i.e., sudden loss of muscle tone typically triggered by emotions), disrupted nighttime sleep, hypnagogic hallucinations, sleep paralysis, and REM sleep behavior disorder [1,2]. NT1 has been associated with reduced quality of life, as well as with occupational, academic, and interpersonal difficulties [3,4,5]. Stigma [4,6] and the frequent co-occurrence of cognitive [7] and psychological disturbances such as depression and anxiety [8,9] further exacerbate the disease burden.

NT1 is caused by a considerable loss of neurons that produce hypocretin-1 (or orexin A) [10,11], which are restricted to the lateral hypothalamus area and project widely throughout the central nervous system [12,13]. The hypocretin system was initially shown to regulate appetite, sleep-wake cycle, and arousal maintenance [14]. However, in the past decade, evidence highlighted that hypocretin neurons regulate a diverse array of behavioral and physiological processes including stress responsivity, emotion regulation, reward-seeking behaviors, and addiction [15,16,17,18,19]. Indeed, the hypocretin neurons send direct and indirect projections to dopaminergic regions (i.e., ventral tegmental area, amygdala, and the nucleus accumbens) [16,17], supporting their role in reward-seeking and addictive behaviors [16,17,20].

Thus, it has been hypothesized that patients with NT1 may be less prone to addiction due to hypocretin cell loss [21,22]. However, available studies found no significant differences between NT1 patients and healthy controls in terms of substance and alcohol abuse, substance addiction [23,24,25], and pathological gambling [26]. In contrast, other findings indicated that patients with NT1 have high levels of other types of addictive behaviors such as binge eating and nocturnal eating [24,27]. To date, no study has addressed addictive online behaviors in NT1 patients.

Addictive online behaviors such as problematic social media use, compulsive Internet use, and problematic online gaming are part of a spectrum of related but discrete Internet-mediated behaviors [28]. Addictive online behaviors are characterized by symptoms typically associated with substance or behavioral addiction such as salience, withdrawal, relapse, tolerance, and mood modification [29,30]. Addictive online behaviors have been conceptualized by the compensatory model of Internet use [31] as “escape behaviors” [32,33] that individuals use as emotion regulation strategies to cope with “offline” negative emotional states [34,35]. Emotion regulation refers to multifaceted processes that involve awareness, understanding, and acceptance of emotions; the ability to control impulsive behaviors and act towards goal-directed behaviors despite experiencing negative emotional states; and the flexible use of situationally appropriate strategies to modulate the intensity and duration of emotional responses, as opposed to eliminating or avoiding emotions [36]. Thus, individuals with impaired emotion regulation may be more prone to regulate negative emotional states such as anxiety and depression by engaging in addictive online behaviors. Several evidences support the validity of the compensatory model of Internet use, highlighting the role of depression [37,38,39,40,41], anxiety [37,38,40,42], and emotion regulation difficulties [43,44,45,46] as consistent predictors and risk factors for addictive online behaviors. Notably, individuals with NT1 have consistently reported higher levels of anxiety [47,48], depression [49,50], and emotion regulation difficulties [51] compared to the general population, which are characteristics that could represent psychological risk factors for the development of addictive online behaviors.

No studies have explored addictive online behaviors in individuals with NT1. Thus, this study aimed to (*i*) evaluate differences in the levels of addictive online behaviors between individuals with NT1 and sex- and age-matched healthy controls (HC); (*ii*) evaluate the association between anxiety, depression, and emotion dysregulation with problematic online gaming, problematic social media use and compulsive Internet use in individuals with NT1.

## 2. Materials and Methods

The Associazione Italiana Narcolettici e Ipersonni (i.e., Italian Association of Patients with Narcolepsy and Hypersomnia, AIN onlus, www.narcolessia.org (accessed on 1 April 2020) publicized an anonymous online survey for patients diagnosed with NT1 at the IRCCS Istituto delle Scienze Neurologiche di Bologna. Data were gathered from April to May 2020 during the first Italian lockdown due to COVID-19. Participants identified sex- and age-matched healthy subjects among friends and/or family members to serve as controls. Inclusion criteria were (*i*) diagnosis of NT1 confirmed by mean latency at the multiple sleep latency test ≤ 8 min with ≥2 sleep onset REM periods and CSF hypocretin deficiency according to *International Classification of Sleep Disorders, 3rd edition*, ICSD-3 [2]; (*ii*) age ≥ 18 years; (*iii*) absence of comorbid neurological and/or psychiatric disorders; and (*iv*) not having received psychological treatment.

The data was gathered using self-administered anonymous questionnaires delivered via an Internet survey conducted using Google Forms. The study was carried out according to the Declaration of Helsinki and was approved by the local ethics committee (Comitato Etico Indipendente di Area Vasta Emilia Centro—CE-AVEC—Prot. N. 66930). Before the start of the survey, each participant provided electronic informed consent and was free to withdraw at any time.

### 2.1. Measures

#### 2.1.1. Sociodemographic Information

The sociodemographic variables evaluated included sex, age, current pharmacological treatment, marital status (single or not), education level (secondary education or higher), and occupation (employed or unemployed).

#### 2.1.2. NT1 Symptoms

Excessive daytime sleepiness was assessed with the Epworth Sleepiness Scale in its Italian version [52], a self-administered questionnaire with 8 items ranging from 0 to 3. The total score ranges from 0 to 24, and higher scores indicate higher level of excessive daytime sleepiness.

Narcoleptic symptoms severity was assessed with the Narcolepsy Severity Scale (NSS) [53]. The NSS is a self-reported questionnaire with 15 items that evaluates the severity, frequency, and impact of the main symptoms of narcolepsy within the last month. It provides three symptom scores: sleep paralysis and hypnagogic hallucinations, excessive daytime sleepiness (EDS), and nighttime sleep and cataplexy. Six items are rated with a 6-point Likert scale ranging from 0 to 5, while nine items are rated with a 4-point scale ranging from 0 to 3. The total score ranges from 0 to 57, with higher scores indicating severe symptoms.

#### 2.1.3. Internet Gaming Disorder Scale—Short Form (IGDS)

The Italian version [54] of the IGDS9-SF [55] was used to assess the severity of problematic online gaming over 12 months. It is a 9-item scale with a 5-point Likert scale ranging from 1 to 5. The items correspond to the nine Internet gaming disorder criteria defined in the American Psychiatric Association’s DSM-5 [56]. Total score ranges from 9 to 45, with higher scores reflecting higher levels of problematic online gaming.

#### 2.1.4. Compulsive Internet Use Scale (CIUS)

Compulsive Internet use was evaluated with the Italian version [57] of CIUS [29]. It comprises 14 items with a 5-point Likert scale ranging from 0 to 4, with the total score ranging from 0 to 56. Higher scores indicate higher levels of compulsive Internet use.

#### 2.1.5. Bergen Social Media Addiction Scale (BSMAS)

Problematic social media use was evaluated with the Italian version [58] of the BSMAS [59]. The scale consists of 6 items with a 5-point Likert scale ranging from 1 to 5, evaluating addictive symptoms such as salience, mood modification, tolerance, withdrawal, conflict, and relapse. The total score ranges from 6 to 30, with higher scores indicating higher levels of problematic social media use.

#### 2.1.6. Difficulties in Emotion Regulation Scale (DERS)

The Italian version [60] of the DERS [36] was used to evaluate emotion regulation. The scale includes 36 items and covers 6 dimensions: non-acceptance (non-acceptance of emotion responses), goals (difficulty in engaging in a goal-directed behavior while experiencing negative emotions), impulse (impulse control difficulty when experiencing negative emotions), awareness (lack of emotional awareness); strategies (limited access to emotion regulation strategies), and clarity (lack of emotional clarity). Items are rated on a 5-point Likert scale ranging from 1 to 5. The total score ranges from 36 to 180, with higher scores indicating greater difficulties with emotion regulation.

#### 2.1.7. Depression Anxiety and Stress Scales (DASS-21)

Depression, anxiety, and stress symptoms were evaluated with the Italian short-form version [61] of the DASS-21 [62]. The depression scale includes items that assess dysphoria, anhedonia, lack of incentive, and low self-esteem; the anxiety scale assesses somatic and subjective symptoms of anxiety and an acute experience of fear; and the stress scale assesses symptoms such as irritability, impatience, tension, and persistent arousal. Each of the three DASS-21 scales contains 7 items on a 4-point Likert scale ranging from 0 to 3. Higher scores indicate higher levels of anxiety, depression, and stress. For this study, scales relative to anxiety and depression were used.

### 2.2. Statistical Analysis

Continuous variables were presented as mean and standard deviation (SD), while categorical variables as absolute (*n*) and relative frequency (%). Due to the unbalanced groups’ sample size, the Welch t-test was used to assess differences between groups in clinical variables [63].

Assumptions of MANCOVA were assessed. The homogeneity of covariances was determined by Box’s M test. Univariate or multivariate outliers were assessed using standardized residuals and Mahalanobis distance values, respectively. The distribution of residuals was evaluated using Shapiro–Wilk’s test. Finally, the presence of a linear relationship between compulsive Internet use, problematic social media use, and problematic online gaming for each group was assessed by visual inspection of a scatterplot.

A one-way MANCOVA was run to determine the effect of group (NT1 vs. HC) on three measures of addictive online behaviors: problematic online gaming, problematic social media use, and compulsive Internet use. The fixed effect of the group (NT1 vs. HC) was controlled for anxiety, depression, and emotion dysregulation to assess if having NT1 was independently associated with addictive online behaviors. We planned to conduct post hoc univariate analyses of variance (ANOVAs) to examine the significant effects. We included anxiety, depression, and emotion dysregulation as covariates in all analyses. Results were considered significant at *p* < 0.05. We used partial eta squared (η^2^*_p_*) as the measure of effect size. Its interpretation is as follows: η^2^*_p_* ≥ 0.01 indicates a small effect, η^2^*_p_* ≥ 0.06 is a medium effect, and η^2^*_p_* ≥ 0.14 is a large effect.

Finally, to evaluate the association between independent variables (i.e., demographic, clinical, and psychological factors) and the outcomes (i.e., addictive online behaviors), three hierarchical multiple regression models were performed for each outcome in the NT1 group. In the first step, demographic and clinical factors were added as covariates. In the second step, psychological factors (i.e., anxiety, depression, and emotion dysregulation) were included to evaluate their unique contribution to explaining the outcome. Results were considered significant at *p* < 0.05. SPSS version 27 was used for all analyses.

## 3. Results

### 3.1. Characteristics of Samples

Forty-three patients with NT1 (mean age 32.21 ± 10.43; range 18–55; 58% females) completed the survey. A total of 1 patient (2%) was drug-free, while 42 patients (98%) were on stable pharmacological treatment at the time of the study, of which 21 were on monotherapy (stimulants, *n* = 12; sodium oxybate, *n* = 8, antidepressant, *n* = 1) and 21 on polytherapy (stimulants and sodium oxybate, *n* = 13; stimulants, sodium oxybate, and antidepressant, *n* = 8). The control group comprised 86 matched participants (mean age 32.02 ± 10.18, range 18–54; 58% females). The Welch t-test results showed a statistically significant difference only in problematic online gaming, with individuals in the NT1 group showing higher levels of problematic online gaming. Clinical characteristics of NT1 patients and the HC group are presented in Table 1.

### 3.2. MANCOVA Results

There was a statistically significant difference between the NT1 and HC group on the combined dependent variables after controlling for anxiety, depression, emotion dysregulation, F(3, 120) = 4.348, *p* < 0.0005, Wilks’ Λ = 0.900, partial η^2^ = 0.098.

Follow-up univariate one-way ANCOVAs were performed. A Bonferroni adjustment was made; thus, statistical significance was accepted at *p* < 0.0167. There were statistically significant differences in adjusted means for IGDS (F(1, 122) = 7.647, *p* < 0.007, partial η^2^ = 0.059).

Pairwise comparisons with a Bonferroni-adjusted *p*-value were made for all three online addictive behaviors measures. Means adjusted for covariates (M_adj_) are presented unless otherwise stated. Scores on IGDS were statistically significantly higher in the NT1 group (M_adj_= 12,487, SE = 0.536) compared to the HC group (M_adj_ = 10.664, SE = 0.377), with a mean difference of 1.823, 95% CI (0.518, 3.128], *p* < 0.005. All other pairwise comparisons were not statistically significant (see Table 2).

### 3.3. Hierarchical Multiple Regression Analysis Results

Hierarchical multiple regression was run to determine if the addition of anxiety, depression, and emotion dysregulation controlling for demographic and clinical factors improved the prediction of the three outcomes. See Table 3 for full details on each regression model.

#### 3.3.1. Compulsive Internet Use

The full model including sex, age, ESS, NSS, DASS-A, DASS-D, and DERS to predict CIUS was not statistically significant, R^2^ = 0.278, F(7, 35) = 1.926, *p* = 0.095; adjusted R^2^ = 0.134. However, the addition of DASS-A, DASS-D, and DERS contributed an additional 24% of the explained variance, ΔR^2^ = 0.245, F(3, 35) = 3.954, *p* < 0.05. Scores on DERS were significantly and positively associated with CIUS scores (see Table 3).

#### 3.3.2. Problematic Social Media Use

The full model including sex, age, ESS, NSS, DASS-A, DASS-D, and DERS to predict BSMAS was statistically significant, R^2^ = 0.375, F(7, 35) = 2.994, *p* < 0.05; adjusted R^2^ = 0.249. The addition of DASS-A, DASS-D, and DERS contributed an additional 32% of the explained variance, ΔR^2^ = 0.320, F(3, 35) = 5.962, *p* < 0.005. DERS scores were significantly and positively associated with BSMAS levels (see Table 4).

#### 3.3.3. Problematic Online Gaming

The full model including sex, age, ESS, NSS, DASS-A, DASS-D, and DERS to predict CIUS was not statistically significant, R^2^ = 0.199, F(7, 35) = 1.238, *p* = 0.309; adjusted R^2^ = 0.038. The addition of DASS-A, DASS-D, and DERS did not lead to a statistically significant increase in the explained variance, ΔR^2^ = 0.103, F(3,35) = 1.506, *p* = 0.230. Sex was significantly associated with IGDS levels (see Table 5).

## 4. Discussion

The purpose of this study was to compare the levels of addictive online behaviors in individuals with NT1 and HC, as well as to identify the clinical and psychological factors associated with addictive online behaviors in NT1 patients.

Our findings showed that individuals with NT1 have significantly higher levels of problematic online gaming than HC. Instead, no effect of the group was found on the levels of compulsive Internet use and problematic social media use, indicating that HC and NT1 patients do not differ significantly.

Similarly to findings from addictive behaviors research in offline contexts, we found an inconsistent association between narcolepsy and addictive online behaviors. For example, Dimitrova et al. reported that substance and alcohol abuse and compulsive gambling were similar between patients with NT1 and HC [24]. In contrast, they found that individuals with NT1 were more prone to binge eating; this finding is in line with other evidence highlighting a higher prevalence of sleep-related eating disorders compared to HC [27]. In addition, Barateau et al. [23] compared the frequency of smoking, alcohol, and substance use in patients with NT1 and HC, finding an increased proportion of both tobacco smoking as well as regular and frequent alcohol consumption habits in individuals with NT1. Heavy drinking, on the other hand, was significantly lower in NT1 patients compared to HC; no significant differences in excessive drug use, substance dependence, or abuse were found between NT1 patients and HC.

It might be speculated that the underlying motivation leading to greater engagement in problematic online gaming in individuals with NT1 stems from their social and interpersonal impairments. Indeed, online gaming spaces can be socially accommodating for individuals with social difficulties, such as NT1 patients. These online spaces indeed provide a variety of social affordances (such as visual anonymity and asynchronicity), which are believed to be especially suitable for socially vulnerable populations, including those that are socially anxious [64]. Up to 53% of narcolepsy patients have been reported to have anxiety disorders such as panic attacks and social phobias [48]. Several factors might contribute to anxiety in individuals with NT1, such as social stigma [4,6], shame [48], fear of falling asleep in front of other people, and unpredictability of cataplectic attacks [65]. Thus, patients with NT1 might use online gaming platforms to fulfill the need for social connections in a ‘‘safe’’ environment and provide the opportunity to engage in social activities in a more comfortable context than face-to-face socialization.

Nevertheless, NT1 patients did not report higher levels of problematic social media use and compulsive Internet use compared to the HC. These results may be related to the age of our sample (mean age 32 years). Indeed, adolescents and young adults are more at risk for compulsive Internet use (e.g., Ref. [39]) and problematic social media use (e.g., Ref. [66]) than older adults.

Sex, age, anxiety, and depression were not significantly associated with compulsive Internet use and problematic use of social media. This is inconsistent with the literature on the general population, which emphasizes a significant association between these variables and addictive online behaviors [40,67].

A significant association between emotion dysregulation, compulsive Internet use, and problematic social media use in NT1 patients was found. This result can be interpreted in light of the compensatory model of Internet use. Individuals with NT1 with difficulties in emotion regulation might engage in problematic use of social media and compulsive Internet use as a coping strategy to manage negative emotional states due to difficulties experienced offline. These findings are consistent with other evidence regarding emotional processing in NT1. Patients with NT1 tend to exhibit impairments in emotional processing, showing lower arousal, attenuated muscular and autonomic to emotional stimuli (evaluated using physiological measures), and also reduced expression of their own emotions (measured through a self-reported questionnaire) [68,69] compared to controls. Interestingly, it has been hypothesized that patients with NT1 inhibited their emotion-expressive behavior in response to emotional stimuli as a compensatory emotion-regulation strategy to reduce or prevent cataplectic attacks [68,69]. Therefore, NT1 patients may have coping mechanisms that alter emotional experiences to avoid cataplexy. Furthermore, sleep and emotion regulation have a bidirectional relationship [70], and poor sleep quality is associated with difficulties in emotion regulation and affective dysfunction [71,72,73]. Notably, disturbed nighttime sleep is a crucial feature of narcolepsy [74] that might negatively affect emotion regulation processes [51].

Emotion dysregulation, depression, and anxiety were not associated with higher problematic online gaming levels. Our findings might indicate that other factors (not examined in our study) might influence this addictive online behavior, such as social difficulties, social anxiety, or lack of connectedness. Future studies are needed to test this hypothesis. Among sociodemographic factors, being male was associated with higher levels of problematic online gaming, which is consistent with previous research that suggests the male sex is a risk factor for developing this behavior [75,76].

Surprisingly, the NT1 and HC groups did not show differences in anxiety and depression levels. This is an unexpected result that is not consistent with previous evidence. Indeed, several studies suggested that individuals with NT1 have higher anxiety and depressive symptoms [5,48]. However, it should be considered that data collection was carried out during the COVID-19 pandemic. Several studies have shown that during the pandemic, the general population reported higher levels of anxiety and depression compared to the pre-pandemic period [77], along with other health consequences [78].

Nevertheless, we can hypothesize that the lockdown and its implications on the social, work-related, and lifestyle domains (e.g., smart working and reduced social activities) may have contributed to reducing anxiety and depression levels. Patients with NT1 could have experienced improved psychological well-being due to reduced social interaction (a trigger for cataplectic attacks) and work activities, allowing a more flexible sleep and nap schedule. This hypothesis is supported by recent evidence according to which NT1 patients teleworking during the pandemic reported decreased sleepiness, cataplexy, fatigue, and higher psychological well-being [79]. Increased nighttime sleep, free napping schedules, and reduced social interaction might have been the mechanisms underlying these improvements [79].

This study suffers from several limitations. One limitation is the small sample size of both the NT1 and HC groups; studies with a larger sample size are required to confirm our findings. A few significant factors were not measured, such as the time of day and the number of hours spent on the Internet, social media, and video games. Moreover, since the research was conducted anonymously by contacting patients through the Italian Association of Patients with Narcolepsy and Hypersomnia, hypocretin levels could not be evaluated; however, 90% of NT1 patients have undetectable hypocretin-1 in cerebrospinal fluid [80,81]. HC were selected by the participant among their social and family members; thus, a selection bias might have occurred. Indeed, addictive behaviors are also influenced by social factors such as level of education and socioeconomic status [82,83,84]; future research could overcome this limitation by randomly selecting HC. Finally, data were collected during the first lockdown due to the COVID-19 spread, which could have affected the results. During the COVID-19-related lockdown, the use of technology increased significantly, with an increased reliance on online devices required for work and educational purposes [85]. Furthermore, due to social isolation and confinement, the use of social media to connect with others, news media consumption, and apps to purchase groceries and other consumer goods have increased [85].

In conclusion, our study showed that addictive online behaviors, specifically problematic online gaming, could be a clinical aspect to consider in individuals with NT1, mainly due to the adverse health (e.g., poor diet, sedentary lifestyle) and psychosocial consequences (e.g., social withdrawal, depression, anxiety) [86,87] of these behaviors, which could worsen the patients’ quality of life. Future research is needed to confirm our results about the levels of addictive online behaviors in NT1 and to determine which potential psychological factors might be associated with problematic online gaming in this population. This could be useful in identifying the most relevant psychological factors for developing effective screening and psychological interventions to reduce addictive online behaviors. Given the specificity of the clinical features of narcolepsy and the disruptive impact on patients’ quality of life and functioning, evidence on this population is needed to develop individualized screening and intervention programs.

## Figures and Tables

**Table 1:** Clinical characteristics of NT1 and HC group.

	NT1		HC		
Variable	Mean ± SD	Range	Mean ± SD	Range	*p*
Excessive daytime sleepiness (ESS)	11.23 ± 4.50	2–23	-	-	
Narcolepsy symptom severity (NSS)	20.51 ± 11.12	5–46	-	-	
Compulsive Internet use (CIUS)	9.40 ± 8.75	0–30	12.27 ± 10.93	0–42	0.110
Problematic social media use (BSMAS)	9.60 ± 3.74	6–18	10.21 ± 4.71	6–24	0.431
Problematic online gaming (IGDS)	12.4 ± 4.59	9–30	10.69 ± 3.03	9–23	0.027
Anxiety (DASS-A)	3.51 ± 3.57	0–14	2.79 ± 3.63	0–18	0.286
Depression (DASS-D)	5.14 ± 5.51	0–20	4.84 ± 4.42	0–21	0.755
Emotion dysregulation (DERS)	79.28 ± 24.05	46–129	83.38 ± 23.52	44–151	0.360

Abbreviations: ESS = Epworth Sleepiness Scale; NSS = Narcolepsy Severity Scale total score; IGDS = Internet Gaming Disorder Scale; CIUS = Compulsive Internet Use Scale; BSMAS = Bergen Social Media Addiction Scale; DERS = Difficulties in Emotion Regulation Scale; DASS-A = Depression Anxiety and Stress Scales—Anxiety scale; DASS-D = Depression Anxiety and Stress Scales–Depression scale.

**Table 2:** Pairwise contrast for adjusted means for the three measures of addictive online behaviors for NT1 and HC group.

Dependent Variable		M_adj_ (95% CI)	Difference in M_adj_ (95% CI)
Compulsive Internet use	NT1	9.641 (7.166,12.115)	−2.504 (−5.551, 0.543)
	HC	12.145 (10.404, 13.885)	
Problematic social media use	NT1	9.617 (8.551, 10.683)	−0.586 (−1.898, 0.727)
	HC	10.203 (9.453, 10.953)	
Problematic online gaming	NT1	12.487 (11.427, 13.547)	1.823 (0.518, 3.128) *
	HC	10.664 (9.918, 11.409)	

Note. * = statistically significant difference (*p* < 0.0167) based on Bonferroni adjustment; 95%.

**Table 3:** Hierarchical multiple regression predicting compulsive Internet use levels.

Dependent Variable						
Compulsive Internet Use	Model 1			Model 2		
	β	SE	*p*	β	SE	*p*
Age	−0.187	0.159	0.330	−0.134	0.146	0.445
Sex	−0.132	2.969	0.441	−0.131	2.728	0.407
Excessive daytime sleepiness	−0.045	0.393	0.825	−0.170	0.363	0.368
Narcolepsy symptom severity	0.106	0.169	0.493	0.009	0.173	0.966
Anxiety				−0.154	0.581	0.521
Depression				0.043	0.371	0.854
Emotion dysregulation				0.577	0.073	0.007
Model Estimates						
*p*	0.857			0.095		
R^2^	0.033			0.278		
F	0.329			1.926		
ΔR^2^				0.245		
ΔF				3.954		

**Table 4:** Hierarchical multiple regression predicting problematic social media use levels.

Dependent Variable						
Problematic Social Media Use	Model 1			Model 2		
	β	SE	*p*	β	SE	*p*
Age	−0.116	0.067	0.541	−0.062	0.058	0.703
Sex	0.079	1256	0.641	0.089	1.086	0.544
Excessive daytime sleepiness	−0.119	0.166	0.555	−0.256	0.144	0.148
Narcolepsy symptom severity	0.265	0.072	0.221	0.155	0.069	0.454
Anxiety				−0.088	0.231	692
Depression				−0.124	0.148	0.574
Emotion dysregulation				0.716	0.029	0.001
Model Estimates						
*p*	0.699			0.014		
R^2^	0.055			0.375		
F	0.551			2.994		
ΔR^2^				0.320		
ΔF				5.962		

**Table 5:** Hierarchical multiple regression predicting problematic online gaming levels.

Dependent Variable						
Problematic Online Gaming	Model 1			Model 2		
	Β	SE	*p*	β	SE	*p*
Age	−0.009	0.081	0.961	0.057	0.081	0.757
Sex	−317	1.508	0.061	−0.363	1.509	0.033
Excessive daytime sleepiness	−0.120	0.200	0.541	−0.151	0.201	0.447
Narcolepsy symptom severity	0.106	0.086	0.614	−0.095	0.096	0.447
Anxiety				0.245	0.322	0.334
Depression				0.024	0.205	0.922
Emotion dysregulation				0.157	0.041	0.464
Model Estimates						
*p*	0.421			0.309		
R^2^	0.095			0.199		
F	0.997			1.238		
ΔR^2^				0.103		
ΔF				1.506		

## Data Availability

Data are available upon request to the corresponding author.
